# Protective Role of Vitamin K3 on SARS-CoV-2 Structural Protein-Induced Inflammation and Cell Death

**DOI:** 10.3390/ph16081101

**Published:** 2023-08-03

**Authors:** Yixiong Zhan, Duoduo Zha, Hongru Lin, Xianxian Mao, Lingyi Yang, Houda Huang, Zongnan He, Sheng Zhou, Fei Xu, Yisong Qian, Yu Liu

**Affiliations:** 1 Pulmonary and Critical Care Medicine, The First Affiliated Hospital of Nanchang University, Nanchang 330006, China; zhanyixiongkfzy@foxmail.com (Y.Z.); xfjxmc@163.com (F.X.); 2 The National Engineering Research Center for Bioengineering Drugs and the Technologies, Institute of Translational Medicine, Nanchang University, Nanchang 330031, China; zdd20170907yx@163.com (D.Z.); 18702519522@hainmc.edu.cn (H.L.); m13549257445@163.com (X.M.); yanglingyiy@163.com (L.Y.); whdhuang@foxmail.com (H.H.); sheng.zhou@se21.qmul.ac.uk (S.Z.); 3 Chongqing Research Institute, Nanchang University, Chongqing 402660, China; 4 Department of Pediatrics, Pingxiang Maternity and Child Care Hospital, Pingxiang 337055, China; hznpxfy@163.com

**Keywords:** SARS-CoV-2, nucleocapsid protein, envelope protein, vitamin K3, inflammation

## Abstract

The structure proteins of severe acute respiratory syndrome coronavirus 2 (SARS-CoV-2), such as nucleocapsid protein (N protein) and envelop protein (E protein), are considered to be the critical pro-inflammatory factors in coronavirus disease 2019 (COVID-19). Vitamin K3 has been reported to exert an anti-inflammatory effect. In this study, we investigated the protective effects of vitamin K3 on SARS-CoV-2 N protein induced-endothelial activation and SARS-CoV-2 E protein induced-cell death in THP-1 cells. The results showed that vitamin K3 reduced N protein-induced monocyte adhesion, suppressed the expression of adhesion molecules, and decreased the mRNA levels of pro-inflammatory cytokines in HLMECs. We confirmed that the effects of vitamin K3 on endothelial activation may be related to the inhibition of the NF-κB signal pathway. In addition, vitamin K3 reversed E protein-induced pyroptosis, inhibited NLRP3/GSDMD signal pathway and reduced the mRNA expression of pro-inflammatory cytokines in THP-1 cells. Our results also showed the protective effects of vitamin K3 on the SARS-CoV-2 structural protein-induced THP-1 cells pyroptosis and endothelial activation via NF-κB signaling pathway. These findings suggested that vitamin K3 potently suppressed the inflammatory response to prevent endothelial activation and monocyte pyroptosis induced by SARS-CoV-2 proteins. This may provide a new strategy for the treatment of COVID-19.

## 1. Introduction

Coronavirus disease 2019 (COVID-19) caused by severe acute respiratory syndrome coronavirus 2 (SARS-CoV-2) has led to a global health crisis [1]. The early symptoms of human infection with SARS-CoV-2 are similar to Middle East respiratory syndrome coronavirus (MERS-CoV), including fever, chills, coughing, malaise, myalgia, and headache. When the disease worsens, SARS-CoV-2 will compromise all systems of the body, leading to acute respiratory distress syndrome (ARDS), systemic inflammatory response syndrome (SIRS), thrombosis, embolism, shock, and ultimately resulting in multi-organ dysfunction [2,3,4]. The worsening of COVID-19 is closely related to vasculopathy, and vascular endothelial inflammation is the cornerstone of organ dysfunction and failure in severe SARS-CoV-2 infection [5,6,7,8].

SARS-CoV-2 is a single-stranded RNA virus that encodes structural and non-structural proteins including spike (S) protein, membrane (M) protein, nucleocapsid (N) protein, envelope (E) protein, and nine auxiliary proteins [9]. SARS-CoV-2 N protein is the central component of virions and restructures viral RNA into ribonucleoprotein complex and promotes viral RNA transcription and replication [10,11]. A case report showed that SARS-CoV-2 N protein present in the brain was associated with endothelial activation, fibrinogen leakage, and immune cell infiltration [12]. Our previous work has found that N protein could serve as a potent inducer of human endothelial activation to specifically upregulate the expression of adhesion molecules and promote monocyte adherence to activated endothelial cells, whereas simvastatin effectively in blocks N protein-induced endothelial activation and inflammation [13]. Thus, the treatment that targets N protein may alleviate SARS-CoV-2-induced endothelial dysfunction and improve the outcome of COVID-19 patients.

SARS-CoV-2 E protein has been reported to mediate inflammatory processes and induce cell death [14,15]. Nucleotide-binding oligomerization domain-like receptor family pyrin domain containing 3 (NLRP3), a macromolecular immune complex, is involved in the extracellular release of IL-1β and IL-18, as well as the cleavage of the pore protein Gasdermin-D (GSDMD). Studies have shown that the NLRP3 inflammasome is active and there are high levels of IL-1β and IL-18 in COVID-19 patients [16,17,18]. SARS-CoV-2 E protein could enhance the NLRP3 inflammasome activation during the later stages of infection [14]. Our previous study also demonstrated that E protein caused pyroptosis in human myeloid leukemia mononuclear (THP-1) cells and contributed to the activation of the NLRP3/GSDMD signal pathway [19]. Therefore, inhibition of NLRP3 activation and GSDMD cleavage may also be a potential therapy for COVID-19.

Vitamin K, as a health-promoting supplement, plays an important regulatory role in inflammation [20]. It has been shown that some vitamin K family members have the potential to reduce COVID-19 mortality and play an important role in SARS-CoV-2-induced lung injury and thromboembolism formation [21,22]. Moreover, vitamin K3 and its analogs can inhibit SARS-CoV-2 3-chymotrypsin-like protease (3CLPro), which plays a crucial role in SARS-CoV-2 transcription and replication [23]. These findings demonstrated that vitamin K may be a promising drug for the treatment of COVID-19. In our previous work, we found that vitamin K3 inhibited monocyte pyroptosis and suppressed systemic inflammation and organ injury in septic mice [24]. According to the above, we hypothesized that vitamin K3 may alleviate the inflammation and organ failure induced by SARS-CoV-2 structural protein.

In this study, we aimed to investigate the protective effects on N protein-induced endothelial activation and E protein-induced cell death, which may provide new insight into the treatment of COVID-19.

## 2. Results

### 2.1. Vitamin K3 Inhibits SARS-CoV-2 N Protein-Induced Endothelial Cell Activation

**Figure 1:**
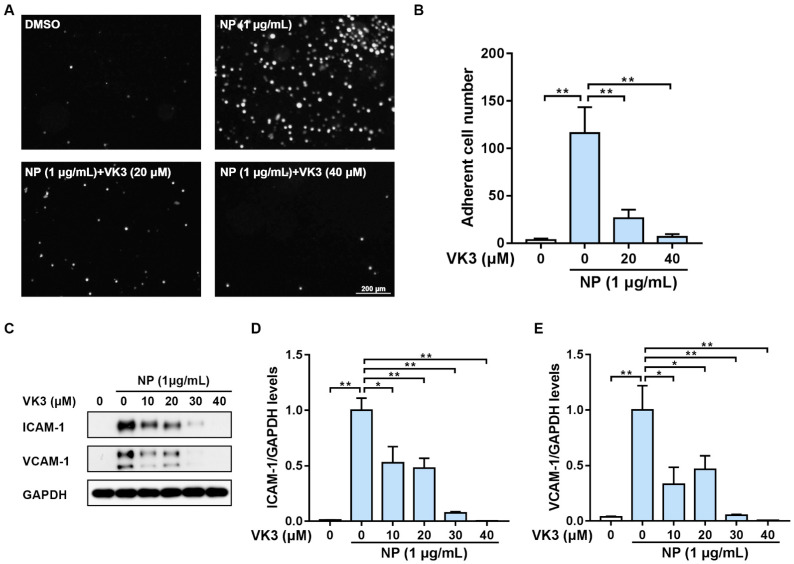
Vitamin K3 suppressed SARS-CoV-2 N protein-induced endothelial activation. HMLECs were treated with vitamin K3 at indicated concentration doses for 1 h with or without subsequent induction of endothelial activation by N protein (1 μg/mL) for 8 h. (**A**) HLMECs were co-cultured with pre-labeled THP-1 cells for 1 h. After washing, the adherent cells were imaged. Scale bars = 200 μm. (**B**) The adherent cell number was quantitatively analyzed. (**C**) The protein levels of ICAM-1 and VCAM-1 were examined by western blot. Quantitative analysis of (**D**) ICAM-1 and (**E**) VCAM-1 was normalized to GAPDH levels and expressed as relative fold changes versus N protein group. Data were shown as means ± SD. * *p* < 0.05 and ** *p* < 0.01.

In our previous work, we found that SARS-CoV-2 N protein specifically induced endothelial activation in HLMECs, which could be reversed by some vitamin K members through screening the small molecule library [13]. Here, we investigated the protective effects of vitamin K3 on endothelial activation. HLMECs were treated with SARS-CoV-2 N protein for 8 h to establish the endothelial activation model. Vitamin K3 at different concentrations was added 1 h before N protein exposure. The adhesion experiment showed that a large number of pre-stained cells (the bright white dots) were present in the N protein-challenged group, whereas 20 μM and 40 μM vitamin K3 treatment significantly reduced the number of adherent THP-1 cells (Figure 1A,B). Western blot was further used to detect the expression of the major cell adhesion molecules, including ICAM-1 and VCAM-1. The protein levels of ICAM-1 and VCAM-1 were significantly increased in HLMECs after N protein exposure. Vitamin K3 ranging from 10–40 μM obviously inhibited the expression of these two adhesion molecules (Figure 1C–E). These results suggested that vitamin K3 potently suppressed N protein-induced endothelial activation in HLMECs.

### 2.2. Vitamin K3 Reduced the Expression of Pro-Inflammatory Cytokines after N Protein Exposure

**Figure 2:**
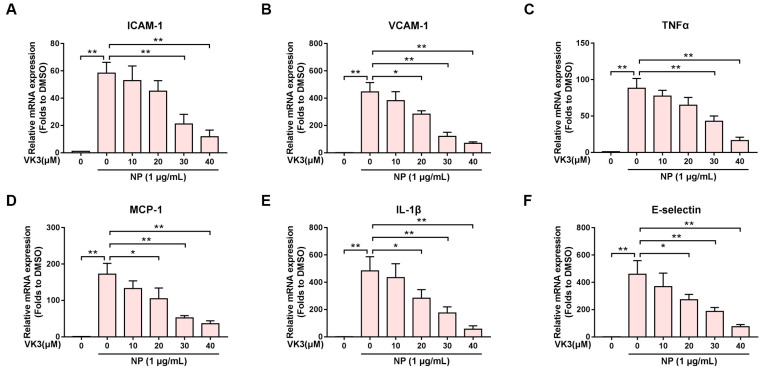
Vitamin K3 reduced the expression of pro-inflammatory cytokines after N protein exposure. HLMECs were pre-treated with indicated concentrations of vitamin K3 for 1 h before N protein (1 μg/mL) exposure for 8 h. The mRNA levels of (**A**) ICAM-1, (**B**) VCAM-1, (**C**) TNF-α, (**D**) MCP-1, (**E**) IL-1β, and (**F**) E-selectin were measured by QPCR. Data were shown as means ± SD. * *p* < 0.05 and ** *p* < 0.01.

To determine whether vitamin K3 inhibits the expression of N protein-induced pro-inflammatory cytokines at the transcriptional level, the real-time quantitative PCR detecting system (QPCR) was used to measure the mRNA levels of the pro-inflammatory cytokines, such as ICAM-1, VCAM-1, TNFα, MCP-1, IL-1β, and E-selectin. In contrast to the control group, the mRNA levels of the pro-inflammatory cytokines were significantly up-regulated in HLMECs treated with N protein. However, vitamin K3 reduced the mRNA expression of these pro-inflammatory factors in a concentration-dependent manner (Figure 2). These results indicated that vitamin K3 attenuated the inflammatory response induced by N protein.

### 2.3. Vitamin K3 Prevents the Activation of NF-κB Signaling Pathway in HLMECs

**Figure 3:**
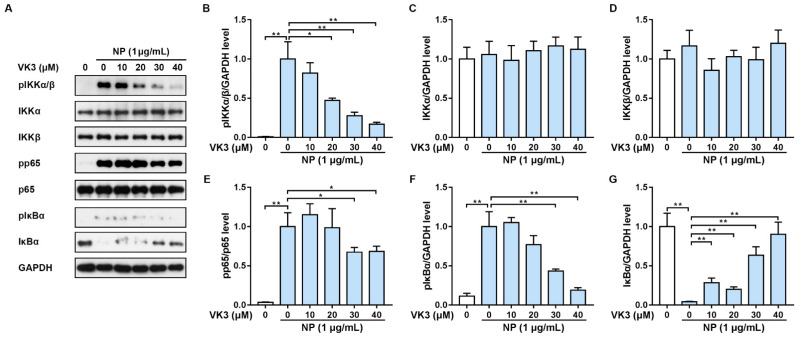
Vitamin K3 inhibited the activation of NF-κB signaling pathway induced by SARS-CoV-2 N protein. HLMECs were pre-incubated with indicated concentrations of vitamin K3 for 1 h before stimulation with N protein (1 μg/mL) for 15 min. (**A**) The protein levels of phospho-IKKα/β (pIKKα/β), IKKα, IKKβ, phospho-p65 (pp65), p65, phospho-IκBα (pIκBα), and IκBα were examined by western blot. Quantitative analyses of (**B**) pIKKα/β, (**C**) IKKα, (**D**) IKKβ, (**F**) pIκBα, (**G**) IκBα were normalized to GAPDH levels and expressed as relative fold changes versus the vehicle group or N protein-treated group. Quantitative analysis of (**E**) pp65 was normalized to p65 levels and expressed as relative fold changes versus N protein-treated group. Data were shown as means ± SD. * *p* < 0.05 and ** *p* < 0.01.

Studies have shown that the NF-κB signaling pathway regulated the expression of ICAM-1 and VCAM-1, which are inextricably linked to endothelial inflammation [25]. According to our previous study, N protein induced endothelial cell activation through the activation of the NF-κB signaling pathway [13]. Therefore, we tried to investigate whether vitamin K3 acts on NF-κB, which in turn affects endothelial activation and monocyte adhesion. HLMECs were pre-treated with vitamin K3 for 1 h followed by N protein exposure. As expected, the phosphorylation of the key components of NF-κB signaling, including pIKKα/β, pp65, and pIκBα, was significantly increased, whereas the levels of total IκBα were decreased in N protein-treated HLMECs, indicating the activation of NF-κB (Figure 3A). In contrast, 20–40 μM vitamin K3 significantly inhibited the phosphorylation of IKKα/β, but did not affect total IKKα or IKKβ levels (Figure 3A–D). Vitamin K3 at 30 and 40 μM effectively reduced the phosphorylation of p65 and pIκBα (Figure 3A,E,F), and all concentrations of vitamin K3 used in the experiment remarkably prevented the N protein-induced degradation of IκBα (Figure 3A,G). These results suggested that vitamin K3 likely suppressed endothelial activation through the modulation of NF-κB activation.

### 2.4. Vitamin K3 Inhibits SARS-CoV-2 E Protein-Induced Pyroptosis in Monocytes

**Figure 4:**
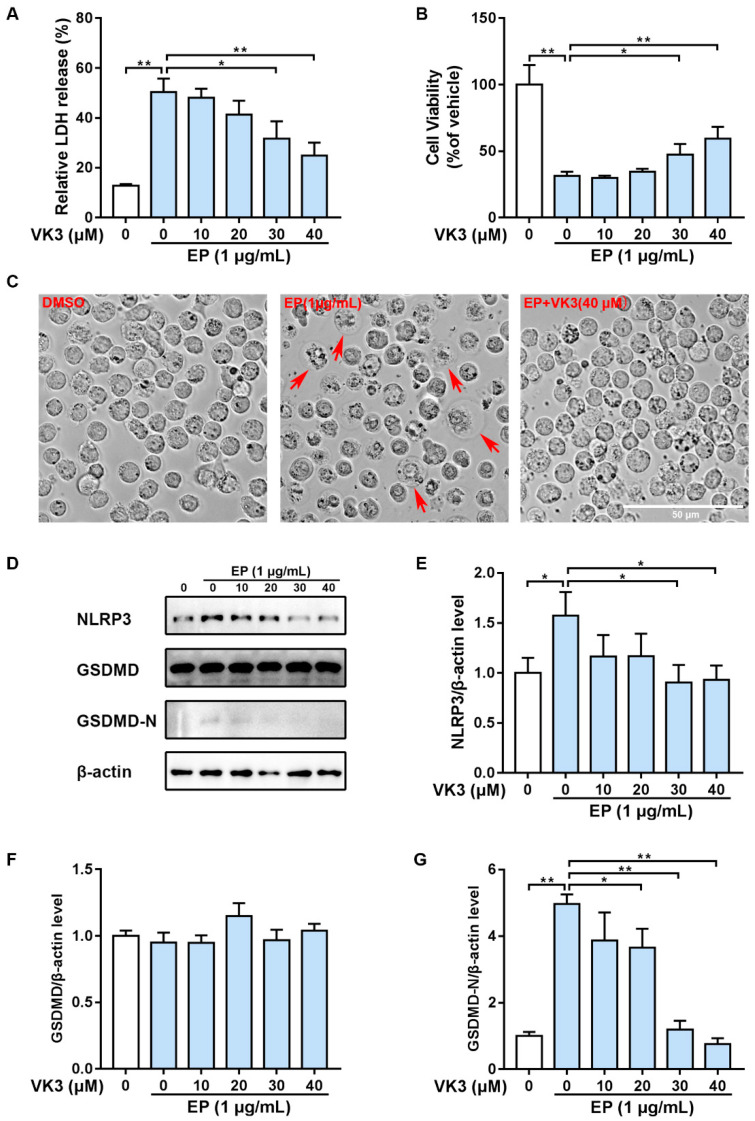
Vitamin K3 attenuated SARS-CoV-2 E protein-induced pyroptosis. THP-1 cells were pre-treated with indicated concentrations of vitamin K3 for 1 h before the induction of SARS-CoV-2 E protein (1 μg/mL). (**A**) The release of LDH to the supernatant and (**B**) the cell viability were both detected using the commercial kits. (**C**) Morphological changes of THP-1 cells were observed under phase contrast microscopy. The pyroptotic cells were indicated by the red arrow. Scale bars = 50 μM. (**D**) The protein levels of NLRP3, GSDMD, and GSDMD-N were examined by western blot. Quantitative analyses of (**E**) NLRP3, (**F**) GSDMD, and (**G**) GSDMD-N were normalized to β-actin levels and expressed as relative fold changes versus the vehicle group. Data were shown as means ± SD. * *p* < 0.05 and ** *p* < 0.01.

Our previous study found that SARS-CoV-2 E protein up-regulated NLPR3 levels and caused pyroptosis in monocytes [19]. We also demonstrated the inhibitory effects of vitamin K3 on LPS plus nigericin-induced pyroptosis [24]. To confirm whether E protein-induced pyroptosis in THP-1 cells is reversed by vitamin K3, different concentrations of vitamin K3 were added to THP-1 1 h before E protein exposure for 4 h. The lactate dehydrogenase (LDH) and 3-(4,5-dimethyl-2-thiazolyl)-2,5-diphenyl-2-H-tetrazolium bromide (MTT) assay showed that E protein resulted in a notable increase in LDH levels in the supernatant and a marked decrease in THP-1 cell viability. Vitamin K3 treatment inhibited the release of LDH and increased the viability of THP-1 cells in a concentration-dependent manner (Figure 4A,B). Morphological observation showed that normal THP-1 cells were round, bright spheres with uniform size and smooth cell edges. In contrast to the control group, cells in the E protein-treated group swelled and ruptured, and the nuclei were shrunken, showing a typical “vacuole-like” pyroptosis morphology, which was significantly reversed by 40 μM of vitamin K3 (Figure 4C).

To evaluate the effect of vitamin K3 on E protein-induced activation of pyroptosis-associated signaling pathway, the expressions of NLRP3 and GSDMD were measured by western blot. It was observed that E protein led to a significant increase in NLRP3 expression and a notable increase in cleaved GSDMD (GSDMD-N) in THP-1 cells (Figure 4D). In contrast, vitamin K3 was found to suppress NLRP3 expression and GSDMD cleavage induced by E protein (Figure 4D–G). Taken together, our results demonstrated that vitamin K3 had a significant inhibitory effect on E protein-induced THP-1 cells pyroptosis through the inhibition of the NLRP3/GSDMD pathway.

### 2.5. Vitamin K3 Decreased the Expression of Pro-Inflammatory Cytokines Induced by E Protein in THP-1

**Figure 5:**
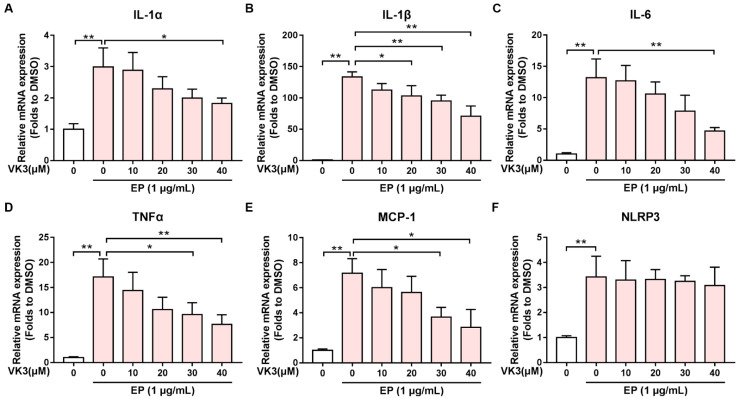
Vitamin K3 reduced the expression of pro-inflammatory cytokines induced by SARS-CoV-2 E protein in THP-1. Vitamin K3 with indicated concentrations was added to THP-1 cells for 1 h before E protein (1 μg/mL) exposure. QPCR was used to measure the mRNA levels of (**A**) IL-1α, (**B**) IL-1β, (**C**) IL-6, (**D**) TNF-α, (**E**) MCP-1, and (**F**) NLRP3. Data were shown as means ± SD. * *p* < 0.05 and ** *p* < 0.01.

Pyroptosis is a form of programmed cell death that occurs in response to infection or injury and is associated with the release of pro-inflammatory cytokines [26]. To investigate whether vitamin K3 regulates the expression of pro-inflammatory cytokines, the mRNA levels of IL-1α, IL-1β, IL-6, TNFα, and MCP-1 were measured via QPCR. The results showed that compared with the control group, the mRNA expressions of the pro-inflammatory cytokines, especially IL-1β, were significantly up-regulated in E protein-treated THP-1 cells, but were significantly inhibited by vitamin K3 (Figure 5A–E). However, E protein-induced up-regulation of NLRP3 was not reversed by vitamin K3 (Figure 5F). The results suggested that vitamin K3 also attenuated E protein-caused excessive inflammatory response in THP-1.

## 3. Discussion

In the present study, we demonstrated the potential protective effects of vitamin K3 on the inflammatory response and cell damage induced by SARS-CoV-2 N protein and E protein. Vitamin K3 suppressed SARS-CoV-2 N protein-induced endothelial cell activation and these effects may be associated with the inhibition of NF-κB signaling pathways. We also found that vitamin K3 prevented SARS-CoV-2 E protein-induced pyroptosis in THP-1 cells, as indicated by up-regulated levels of NLRP3 and cleaved GSDMD. Additionally, vitamin K3 is potent in inhibiting the expression of pro-inflammatory cytokines stimulated by SARS-CoV-2 proteins in endothelial cells and monocytes, respectively. This study illustrated the inextricable relationship between inflammation and SARS-CoV-2 proteins and suggested that vitamin K3 may have potential as a preventive or therapeutic strategy to combat inflammation and organ injury in COVID-19. Endothelial activation is a key feature of the inflammatory response in COVID-19, and is associated with increased vascular permeability, microvascular thrombosis, and organ failure [27].

Vitamin K plays a crucial biological role in maintaining normal blood coagulation, bone mineralization, soft tissue physiology, and neurological development [28]. Vitamin K is not a single compound; rather, it is a general term covering natural plant and animal forms (vitamin K1 and K2) and their synthetic congeners (vitamin K3 and K4) with almost no reported cases of systemic toxicity [29]. Vitamin K3, also known as Menadione, is hydrophilic and not obtained through the diet [29]. It is the simplest form of vitamin K and can always be used as a clotting drug and vitamin supplement [23]. In recent years, the anti-inflammatory effect of vitamin K3 has been reported widely [30]. Kiely et al. showed that vitamin K potentially improved cognitive function by inhibiting the NF-κB activation and pro-inflammatory cytokine production [31]. In other studies, vitamin K3 and its analogs may exert their inhibitory effect on inflammation by targeting the NLRP3 inflammasome and inhibiting the NF-κB signaling pathway [24,32]. It has been reported that there is increased vitamin K utilization in COVID-19 patients, and that low vitamin K could predict higher mortality among the COVID-19 patients [33,34]. Moreover, Vitamin K3 and its analogs have been confirmed to inhibit the activity of SARS-CoV-2 3CL^Pro^ and serve as a potential compound to combat COVID-19 [23]. However, the mechanism underlying the protective activity of vitamin K remains largely unknown. Therefore, it is meaningful work to study the effect of vitamin K3 on SARS-CoV-2-induced inflammation and injury and explore its mechanism of action.

The structural proteins of SARS-CoV-2 play complex roles in virus-induced inflammation and damage. SARS-CoV-2 N protein, as one of the most crucial structural components that bind to the genome, plays an important role in protecting the viral genome [35]. Our previous study found that SARS-CoV-2 N protein-induced endothelial activation could be reversed by some small molecules, including the vitamin K family [13]. Here, we confirmed that vitamin K3 effectively suppressed N protein-induced endothelial activation through inhibiting monocyte adherence and reducing the expression of the key adhesion molecules. In addition, vitamin K3 exerted potently anti-inflammatory activity to prevent the expression of multiple pro-inflammatory cytokines. These effects may be related to the inhibition of the major pro-inflammatory signal pathway NF-κB. These results suggested that as an anti-inflammatory compound, vitamin K3 may have beneficial effects on SARS-CoV-2-induced endothelial activation and dysfunction during the pathogenesis of COVID-19.

Pyroptosis is a form of programmed cell death triggered by proinflammatory signals, and includes cell swelling and ballooning [36]. In the classical pyroptosis pathway, the NLRP3 inflammasome can bind to the pyrin domain (PYD) of apoptosis-associated speck-like protein containing a caspase recruitment domain (ASC) through its N-terminal PYD. Then, the protein complex recruits and combines with po-caspase-1 through the caspase recruitment domain (CARD) and leads pro-caspase to mature by self-cleavage [37]. Then, the inflammasome complexes are formed and can convert cytokine precursors to their mature forms, and induce GSDMD to undergo cleavage [17]. The cleaved GSDMD-N-terminal binds to the membrane lipids and induces cell perforation, leading to cell rupture and death [38]. Pyroptosis was found in COVID-19 patients with increases of IL-1β and IL-18 [39], which further promoted the secretion of pro-inflammatory cytokines such as IL-6, IFN-γ, MCP-1/CCL2, MIP-1α/CCL3, MIP-1β/CCL4 [39,40,41]. These secreted cytokines subsequently recruited activated macrophages and T-cells to the sites of infection and exacerbated the inflammatory response. Meanwhile, it has also been shown that vitamin K analogs can inhibit inflammation by targeting NLRP3 [32]. One study has shown that SARS-CoV-2 E protein could induce rapid cell death in various susceptible cell types, and can promote robust secretion of cytokines and chemokines in macrophages [15]. We recently demonstrated that SARS-CoV-2 E protein activated NF-κB, JNK, and p38 pathways, thereby resulting in pyroptosis and the release of pro-inflammatory cytokines in THP-1 cells [19]. In the manuscript, we found that vitamin K3 significantly improved E protein-induced THP-1 pyroptosis and inhibited the classic NLRP3/GSDMD pathway. It was reported that NF-κB is an important transcription factor of GSDMD, which is defined as a key effector of pyroptosis [42]. Numerous studies have also shown a regulatory relationship between NF-κB and the activation of NLRP3 and GSDMD [43]. According to the previous studies and our work, vitamin K3 inhibited pyroptosis by suppressing the activation of NF-κB [24]. Therefore, the protective effect of vitamin K3 against E protein-induced pyroptosis may also be associated with the inhibition of NF-κB.

It is noted that our results showed that vitamin K3 did not affect the mRNA expression, but did significantly reduce the protein level of NLRP3, indicating that vitamin K3 may regulate its expression in a post-transcriptional manner. This suggested that vitamin K3 may play a protective role by targeting and down-regulating NLRP3, thereby reducing inflammasome assembly and GSDMD cleavage. However, the detailed mechanism warranted further investigation in our lab. In addition, since mice are less vulnerable to SARS-CoV-2 challenge [44]. We are now trying to establish other animal models to further verify the effects of vitamin K3 in response to SARS-CoV-2 proteins in vivo.

## 4. Materials and Methods

### 4.1. Reagents

SARS-CoV-2 nucleocapsid proteins and envelope proteins were obtained from ACRO Biosystems (Beijing, China). Vitamin K3 was purchased from Solarbio (Beijing, China). RPMI-1640 medium and fetal bovine serum were obtained from Gibco (Waltham, MA, USA). NLRP3 and NF-κB signaling pathway antibodies were obtained from Cell Signaling Technology (Danvers, MA, USA). GSDMD and ICAM-1 antibodies were purchased from Proteintech (Wuhan, China). VCAM-1 and β-actin antibodies were purchased from Santa Cruz Biotechnology, Inc. (Dallas, TX, USA). Goat Anti-Rabbit IgG (H + L) secondary antibody and Goat Anti-Mouse IgG (H + L) secondary antibody were from Thermo (Waltham, MA, USA).

### 4.2. Cell Culture and Treatment

HLMECs were obtained from Lonza Bioscience (Houston, TX, USA) and were cultured in EGMTM −2 MV Microvascular Endothelial Cell Growth Medium (Lonza Bioscience). HLMECs were pre-treated with various concentrations of vitamin K3 for 1 h, followed by the exposure of 1 μg/mL SARS-CoV-2 N protein for 8 h. THP-1 cells were cultured in RPMI1640 medium in a humidified incubator at 37 °C with 5% CO2. THP-1 cells were pre-treated with Vitamin K3 for 1 h and then exposed to 1 μg/mL SARS-CoV-2 E protein for 4 h.

### 4.3. Monocyte Adhesion Assay

THP-1 cells were pre-labeled with PKH67 fluorescent staining dye (Zynaxis) in RPMI1640 culture medium for 30 min, and then incubated with HLMECs for 1 h. HLMECs were gently washed with cold RPMI1640 culture medium to remove unadhered cells. The adhered THP-1 was observed and the images were taken using the Cytation 3 cell imaging multi-mode reader.

### 4.4. LDH Assay

The release of LDH into the supernatant was detected by CytoTox 96 Non-Radioactive Cytotoxicity Assay Kit (Promega, Madison, WI, USA). THP-1 in the 96-well plates was centrifuged at 1000 rpm for 5 min. The supernatant was transferred to another 96-well plate. A large amount of Assay buffer was added to each well and incubated for 20 min at room temperature and protected from light. The stop buffer was then added to each well to terminate the reaction. The optical density (OD) value was read at 490 nm for the following calculation: LDH leakage rate (%) = (OD lysate group − OD blank)/ (OD experimental group − OD blank) × 100%.

### 4.5. MTT Assay

Cell viability was analyzed using the MTT colorimetric method. At the end of treatment, THP-1 cells were incubated with MTT for 4 h in the dark at 37 °C. The succinate dehydrogenase in the mitochondria reduces exogenous MTT to water-insoluble blue-purple crystalline formazan in living cells. The formazan was dissolved by Dimethyl sulfoxide (DMSO), and its optical density value was measured at 570 nm by the microplate reader.

### 4.6. QPCR

The total RNA was extracted from cells using the RNasy Mini Kit (Qiagen, Germantown, MD, USA). The first-strand cDNAs were synthesized by using High-Capacity RNA-to-CDNA Kit (Thermo Fisher Science, Vilnius, Lithuania). The reaction system consisted of 2 × SYBR Green PCR Master Mix (Applied Biosystems, Foster City, CA, USA), primer pairs, and cDNA. The reactions consisted of a two-step thermal cycling (95 °C for 15 s and 60 °C for 1 min; 40 cycles). Data were analyzed using ABI detection system software, through the 2^−ΔΔCT^ method. The primer sequences used were listed in Table 1.

### 4.7. Western Blot

The total protein was collected with ice cold RIPA lysis buffer. Equal amounts of protein were added to an SDS-PAGE gel and separated by gel electrophoresis. Then, the electrophoresed proteins were transferred to the nitrocellulose (NC) membrane. Non-specific binding was blocked with 5% (*w*/*v*) skim milk. After blocking, the membranes were incubated overnight at 4 °C with primary antibodies. Next, the membranes were incubated with HRP-conjugated secondary antibodies at room temperature for 1.5 h. Luminescence was generated after the membranes were exposed to Super Signal West Pico Chemiluminescent Substrate (Thermo Fisher Scientific) and was detected using the ChemiDoc^TM^ Imaging System (Bio-Rad, Hercules, CA, USA).

### 4.8. Statistical Analysis

All experimental data was repeated at least three times and expressed as mean ± standard deviation (mean ± SD). Statistical differences between two groups were analyzed by *t*-test for comparison, and statistical differences between multiple groups was analyzed by One-way ANOVA. Difference of *p* < 0.05 was considered statistically significant.

## 5. Conclusions

In summary, we confirmed that vitamin K3 has beneficial effects on both SARS-CoV-2 N protein-induced endothelial activation and E protein-induced pyroptosis in monocytes. The protective effects of vitamin K3 on SARS-CoV-2 protein-induced excessive inflammatory response may all be related to the NF-κB signaling pathway. Moreover, the protective effect of vitamin K3 on E protein-induced cell death may be achieved by targeting NLRP3, thereby inhibiting GSDMD cleavage. The results provide a basis for vitamin supplementation as a key drug in clinical treatment or improvement of COVID-19 outcomes in the future.

## Figures and Tables

**Table 1:** Primers used in QPCR reactions.

Gene	Forward Primer (5′→ 3′)	Reverse primer (5′ → 3′)
Human ICAM-1	AGCTTCGTGTCCTGTATGGC	TTTTCTGGCCACGTCCAGTT
Human VCAM-1	TGTTTGCAGCTTCTCAAGCTTTT	GATGTGGTCCCCTCATTCGT
Human TNF-α	TCTCGCACCCCGAGTGA	GGAGCTGCCCCTCAGCTT
Human MCP1	CAGCCAGATGCAATCAATGCC	TGGAATCCTGAACCCACTTCT
Human IL-1α	AGATGCCTGAGATACCCAAAACC	CCAAGCACACCCAGTAGTCT
Human IL-1β	CCACAGACCTTCCAGGAGAATG	ATCCCATGTGTCGAAGAAGATAGG
Human IL-6	CCTGAACCTTCCAAAGATGGC	TTCACCAGGCAAGTCTCCTCA
Human IL-18	GCATCAACTTTGTGGCAAT	CCGATTTCCTTGGTCAAT
Human NLRP3	CGTGAGTCCCATTAAGATGGAGT	CCCGACAGTGGATATAGAACAGA
Human E-Selectin	GGCAGTTCCGGGAAAGATCA	GTGGGAGCTTCACAGGTAGG

## Data Availability

Data generated or analyzed during this study are available from the corresponding author upon reasonable request.
